# Classifying and communicating risks in prediabetes according to fasting glucose and/or glycated hemoglobin: PREDAPS cohort study

**DOI:** 10.1080/02813432.2021.1958497

**Published:** 2021-08-04

**Authors:** Enrique Regidor, Lucía Cea-Soriano, Antonio Ruiz, Albert Goday, David Carabantes, Javier Díez-Espino, Sara Artola, Josep Franch-Nadal, for the PREDAPS Study Group

**Affiliations:** a Department of Public Health and Maternal and Child Health, Faculty of Medicine, Universidad Complutense de Madrid, Madrid, Spain; b Instituto de Investigación Sanitaria del Hospital Clínico San Carlos (IdISSC), Madrid, Spain; c CIBER Epidemiología y Salud Pública (CIBERESP), Madrid, Spain; d redGDPS Foundation, Madrid, Spain; e Centro de Salud Universitario Pinto, Madrid, Spain; f Universidad Europea de Madrid, Madrid, Spain; g Servicio de Endocrinología, Hospital del Mar, IMIM, Barcelona, Spain; h Department of Medicine, Universitat Autònoma de Barcelona, Barcelona, Spain; i CIBER Fisiopatología de la Obesidad y Nutrición, Madrid, Spain; j Tafalla Health Center, Navarra, Spain; k Instituto de Investigación Sanitaria de Navarra (IDiSNA), Pamplona, Spain; l Centro de Salud José Marvá, Madrid, Spain; m Barcelona City Research Support Unit, University Institute for Research in Primary Care Jordi Gol, Barcelona, Spain; n CIBER Diabetes y Enfermedades Metabólicas Asociadas, Madrid, Spain; o Department of Medicine, Universidad de Barcelona, Barcelona, Spain

**Keywords:** Prediabetes, absolute risk, odds, progression, cohort study

## Abstract

**Objective:**

Information about prognostic outcomes can be of great help for people with prediabetes and for physicians in the face of scientific controversy about the cutoff point for defining prediabetes. We aimed to estimate different prognostic outcomes in people with prediabetes.

**Design:**

Prospective cohort of subjects with prediabetes according to American Diabetes Association guidelines.

**Main Outcome measures:**

The probabilities of diabetes onset versus non-onset, the odds against diabetes onset, and the probability of reverting to normoglycemia according to different prediabetes categories were calculated.

**Results:**

The odds against diabetes onset ranged from 29:1 in individuals with isolated FPG of 100–109 mg/dL to 1:1 in individuals with FPG 110–125 mg/dL plus HbA1c 6.0–6.4%. The probability of reversion to normoglycemia was 31.2% (95% CI 24.0–39.6) in those with isolated FPG 100–109 mg/dL and 6.2% (95% CI 1.4–10.0) in those with FPG 110–125 mg/dL plus HbA1c 6.0–6.4%. Of every 100 participants in the first group, 97 did not develop diabetes and 31 reverted to normoglycemia, while in the second group those figures were 52 and 6.

**Conclusions:**

Using odds of probabilities and absolute numbers might be useful for people with prediabetes and physicians to share decisions on potential interventions.

**Key points**
- Communicating knowledge on the course of the disease to make clinical decisions is not always done appropriately.
- Prediabetes is an example where risk communication is important because the prognosis of subjects with prediabetes is very heterogeneous.
- Depending on fasting plasma glucose and HbA1c levels, the odds of probabilities against diabetes onset ranged from 29: 1 to 1: 1.
- Depending on fasting plasma glucose and HbA1c levels, the number of subjects in 100 who revert to normoglycemia ranged from 31 to 6.
- Using probabilities and number absolutes on the prognosis of prediabetes may be useful for people with prediabetes and physicians to share decisions on potential interventions.

## Introduction

Identifying patients at risk of developing a disease in order to implement interventions that prevent or delay onset is a key activity of clinical practice. Early identification of these individuals requires knowledge of the natural history of the disease and empirical evidence that supports the effectiveness of the intervention [1–2]. The information gleaned from this process must then be communicated to patients, enabling them to make an informed decision about potential interventions. However, communicating knowledge on the course of the disease is not always done appropriately. Instead, individuals with a pathological or metabolic anomaly frequently hear that they are at higher risk for developing the disease [3–4]. This information may be insufficient for making a decision, as a situation that doubles the risk of developing the disease could reflect an absolute increase of 10 to 20%, or of 0.1 to 0.2%.

One option is to prioritize communication on absolute risk [5–7]. This strategy may sometimes require knowledge on the magnitude of the risk of developing the diseases for a variety of values, for example, when the range of abnormal metabolic values (above normal but below the diagnostic threshold) is wide. With this information, people situated at different points along this range could better understand their chances of developing the disease. Similarly, in situations in which metabolic indicators could revert to normal, it is necessary to communicate the likelihood of this happening.

Prediabetes is a case in point. The term refers to situations in which blood glucose or glycated hemoglobin (HbA1c) is higher than normal but lower than the cutoff for diabetes diagnosis [8–9]. People with prediabetes present a higher risk of developing diabetes than those without [8–10], but some also revert to normoglycemia [9,11,12]. Thus, in people with different levels of prediabetes, communication of this information could be useful for deciding between different strategies to lower the risk of diabetes or raise the probability of returning to normal glycemic levels.

This informed decision-making could also be of great help to clinicians given the controversy among the scientific community with regard to the lower limit of fasting plasma glucose (FPG) and HbA1c for defining prediabetes [13–17]. The American Diabetes Association (ADA) established that a subject with FPG between 100 and 125 mg/dL or with HbA1c between 5.7% and 6.4% has prediabetes [8]. According to some authors, physicians should propose weight loss or physical exercise, to delay the development of diabetes, to subjects with these a FPG or HbA1c values. And, in some cases, incorporate pharmacological therapy [13–15]. However, other authors consider that these values represent an unnecessary burden for the health system, since most of these subjects will not develop diabetes in at least 10 years of follow-up and a large part will revert to normoglycemia [16–18]. In addition, these subjects will experience the secondary effects of the interventions without this early identification implying an improvement in their prognosis.

The aim of this study, performed in a prospective cohort of people with prediabetes according to American Diabetes Association (ADA) criteria, is to estimate several prognostic outcomes according to a wide range of FPG and/or HbA1c values and to show the results as odds of probabilities and as absolute numbers to aid patients and their physicians in decision-making.

## Subjects, material and methods

### Study design

The Primary Health Care Study on the Evolution of Patients with Prediabetes (PREDAPS Study) followed a cohort of people with prediabetes and another without glucose metabolism disorders. Details of the study have been described previously [19]. Family physicians throughout Spain conducted the study in the context of everyday clinical practice. In 2012, people aged 30 to 74 years who presented to primary health care for any reason were invited to participate in the study. Exclusion criteria were: diabetes, terminal disease, pregnancy, surgery or hospital admission in the three months prior to baseline, or any hematological disease which could alter HbA1c values. Analyses were restricted to the prediabetes cohort. Individuals were included in this cohort if they met ADA criteria for prediabetes: FPG levels of 5.5 to 6.9 mmol/L (100 mg/dL to 125 mg/dL) and/or HbA1c range from 39 to 47 mmol/mol (5.7% to 6.4%). In 2012, 1184 people with prediabetes were enrolled; 831 completed the five years of follow-up to 2017. Therefore, 353 subjects dropped out of the study before 2017. These subjects were included in the analyzes and contributed to the risk of diabetes and the likelihood of reverting to normoglycemia up to the time of loss to follow up. Mean follow-up was 4.2 years.

Participants gave their written informed consent before inclusion. The study was classified by the Spanish Agency of Medicines and Medical Devices as a non-intervention (observational) post-authorization study, and the study protocol was approved by the Parc de Salut Mar Clinical Research Ethics Committee in Barcelona (2011-4274-I).

### Procedure

After collecting baseline information about sociodemographic characteristics, medical history, and health-related habits from participants’ clinical records, their family physicians conducted personal interviews. FPG and HbA1c values were obtained annually. Participants were followed for up to five years or until dropout, death, or diabetes onset.

Diabetes was defined as either FPG ≥7 mmol/L (126 mg/dL) or HbA1c ≥48 mmol/mol (6.5%). Reversion to normal glucose regulation was considered if FPG and HbA1c values were FPG < 5.5 mmol/L (100 mg/dL) and HbA1c <39 mmol/mol (5.7%), respectively, at the time of the last visit.

For this study, participants were divided into eight categories on the basis of baseline diagnosis of prediabetes: isolated FPG 5.5-6.0 mmol/L (100–109 mg/dL), isolated FPG 6.1–6.9 mmol/L (110–125 mg/dL), isolated HbA1c 39–41 mmol/mol (5.7–5.9%), isolated HbA1c 42–47 mmol/mol (6.0–6.4%), FPG 5.5–6.0 mmol/L (100–109 mg/dL) plus HbA1c 39–41 mmol/mol (5.7–5.9%), FPG 5.5–6.0 mmol/L (100–109 mg/dL) plus HbA1c 42–47 mmol/mol (6.0–6.4%), FPG 6.1–6.9 mmol/L (110–125 mg/dL) plus HbA1c 39–41 mmol/mol (5.7–5.9%), and FPG 6.1–6.9 mmol/L (110–125 mg/dL) plus HbA1c 42–47 mmol/mol (6.0–6.4%).

### Statistical analysis

For each prediabetes category, the sex- and age-adjusted incidence rate of diabetes and the sex- and age-adjusted incidence rate of regression to normoglycemia per 1000 person-years were calculated, with 95% confidence intervals (CI) [20]. The standard population for adjustment (distribution by age and sex) was the entire prediabetes cohort. Based on these incidence rates, the probability of diabetes onset or reverting to normoglycemia during follow-up were calculated. Incidence rates were assumed to remain constant over five years of follow-up, such that each probability has been estimated as 1 – exp (−incidence rate * 5) [21]. Likewise, based on these probabilities, for every 100 people in each prediabetes category, the number of participants who developed diabetes, reverted to normoglycemia, and continued at prediabetes levels were calculated (the latter was calculated by subtracting the sum of the two previous proportions from the unit) and represented by pictograms.

The probability of diabetes non-onset was calculated in a complementary way as the probability of diabetes onset. Since diabetes non-onset can be considered a successful outcome, we also calculated the odds between the probability of diabetes non-onset versus onset. For example, an odds of 3 (3:1) would mean that diabetes non-onset is three times more likely than diabetes onset. And after converting the odds into probability, that is, dividing the odds by one and then adding the odds, it would mean that 3 out of 4 people would not develop diabetes.

Finally, additional analyses were used to test the extent to which the differences in prognosis between prediabetes categories could be due to differences in health behaviors. Cox regression analysis was used to determine the magnitude of the association of prediabetes categories with the risk of diabetes onset and with reversion to normoglycemia. The first model included adjustment for sex and age. In the second, health behaviors at baseline were added to model 1. And in the third model, health behaviors assessed at each year of follow-up were entered as time-dependent covariates and added to model 1.

## Results

Supplementary table 1 shows the baseline characteristics—demographic distribution, biochemical parameters, hypertension, obesity and health-related behaviors—according to each category of prediabetes. Table 1 shows the number of participants at baseline, person-years of follow-up, and sex and age-adjusted incidence rates. The overall incidence rates of diabetes and reversion to normoglycemia were 42.8 per 1000 person-years (95% CI 37.0–48.6) and 43.9 per 1000 person-years (95% CI 38.1–49.8), respectively. The lowest incidence rate of diabetes was observed in the prediabetes category for isolated FPG 100–109 mg/dL; and the highest, in individuals with FPG 110–125 mg/dL plus HbA1c 6.0–6.4%. As for reversion to normoglycemia, the highest rate was in the group with isolated HbA1c 5.7–5.9%, and the lowest in the category with FPG 110–125 mg/dL plus HbA1c 6.0–6.4%.

**Table 1.** People at the beginning of follow-up, person-years of follow-up, and sex and age-adjusted incidence rates of diabetes and regression to normoglycemia per 1000 person-years according prediabetes category.

Prediabetes category	People at the beginning of follow-up	Person-years of follow-up	Incidence rate of diabetes (95% CI)		Incidence rate of regression (95% CI)	
Isolated FPG 100–109 mg/dL	152	680	6.8	(0.6–13.0)	74.8	(55.0–100.9)
Isolated FPG 110–125 mg/dL	102	426	38.4	(20.5–56.3)	36.2	(19.3–55.8)
Isolated HbA1c 5.7–5.9%	204	941	9.9	(3.7–16.1)	84.5	(66.2–103.9)
Isolated HbA1c 6.0–6.4%	112	501	19.3	(6.5–32.2)	38.7	(5.9–50.0)
FPG 100-109 mg/dL & HbA1C 5.7–5.9 %	138	600	35.2	(18.6–51.8)	29.0	(16.4–43.6)
FPG 100-109 mg/dL & HbA1C 6.0–6.4 %	128	524	52.8	(30.3–75.3)	23.2	(5.3–36.7)
FPG 110-125 mg/dL &HbA1C 5.7–5.9 %	140	579	55.5	(36.1–74.8)	30.3	(14.6–44.1)
FPG 110-125 mg/dL &HbA1C 6.0–6.4 %	208	671	132.3	(103.9–160.8)	12.9	(2.8–21.0)
Overall	1184	4922	42.8	(37.0–48.6)	43.9	(38.1–49.8)

95% CI: 95% confidence interval.

Table 2 shows the probability of diabetes non-onset versus onset, as a percentage, and the odds against diabetes onset according to the prediabetes categories. The overall probabilities of diabetes non-onset versus onset were 80.7% vs 19.3%, and the overall odds were 4:1. The highest odds against diabetes onset were observed in individuals with isolated FPG 100-109 mg/dL (29:1) and isolated HbA1c 5.7–5.9% (20:1). Their probabilities of diabetes non-onset versus onset were, for the isolated FPG 100–109 mg/dL group, 96.7% (95% CI 93.7–99.7) vs 3.3% (95% CI 0.3–6.3); and for the isolated HbA1c 5.7–5.9% group, 95.2% (95% CI 92.3–98.2) vs 4.8% (95% CI 1.8–7.7). The lowest odds against diabetes onset were observed in the FPG 110–125 mg/dL plus HbA1c 6.0–6.4% group (1:1), whose probabilities of diabetes non-onset versus onset were 51.6% (95% CI 44.8–59.5) vs 48.4% (95% CI 48.5–55.2).

**Table 2.** Sex and age-adjusted probabilities of diabetes non-onset and onset, odds of diabetes non-onset versus onset and sex and age-adjusted probability of reversion to normoglycemia.

Prediabetes category	Diabetes						
	Probability (%) of diabetes non-onset (95% CI)		Probability (%) of diabetes onset (95% CI)		Odds of non-onset versus onset (95% Cl)	Probability (%) of reversion to normoglycemia (95% CI)	
Isolated FPG 100–109 mg/dL	96.7	(93.7–99.7)	3.3	(0.3–6.3)	29: 1	31.2	(24.0–39.6)
Isolated FPG 110–125 mg/dL	82.5	(75.5–90.3)	17.5	(9.7–24.5)	5: 1	16.6	(9.2–24.3)
Isolated HbA1c 5.7–5.9%	95.2	(92.3–98.2)	4.8	(1.8–7.7)	20: 1	34.5	(28.2–40.5)
Isolated HbA1c 6.0–6.4%	90.8	(85.1–96.8)	9.2	(3.2–14.9)	10: 1	17.6	(2.9–22.1)
FPG 100–109 mg/dL & HbA1C 5.7–5.9 %	83.9	(77.2–91.1)	16.1	(8.9–22.8)	5: 1	13.5	(7.9–19.6)
FPG 100–109 mg/dL & HbA1C 6.0–6.4 %	76.8	(68.8–83.5)	23.2	(14.1–31.4)	3: 1	11.0	(2.6–16.8)
FPG 110–125 mg/dL &HbA1C 5.7–5.9 %	75.8	(68.6–85.9)	24.2	(14.1–31.4)	3: 1	14.1	(7.0–19.8)
FPG 110–125 mg/dL &HbA1C 6.0–6.4 %	51.6	(44.8–59.5)	48.4	(40.5–55.2)	1: 1	6.2	(1.4–10.0)
Overall	80.7	(78.4–83.1)	19.3	(16.9–21.6)	4: 1	19.7	(17.3–22.0)

95% CI: 95% Confidence interval. PREDAPS Study. 1. Probability of diabetes non-onset versus onset.

Table 2 also shows the probability (in percentage) of reversion to normoglycemia. The overall probability was 19.7% (95% CI 17.3-22.0). The highest probabilities were among those with isolated HbA1c 5.7–5.9% (34.5%, 95% CI 28.2–40.5) and isolated FPG 100–109 mg/dL (31.2%, 95% CI 24.0–39.6), while the lowest was in the category with FPG 110–125 mg/dL plus HbA1c 6.0–6.4% (6.2%, 95% CI 1.4–10.0).

**Figure 1.**
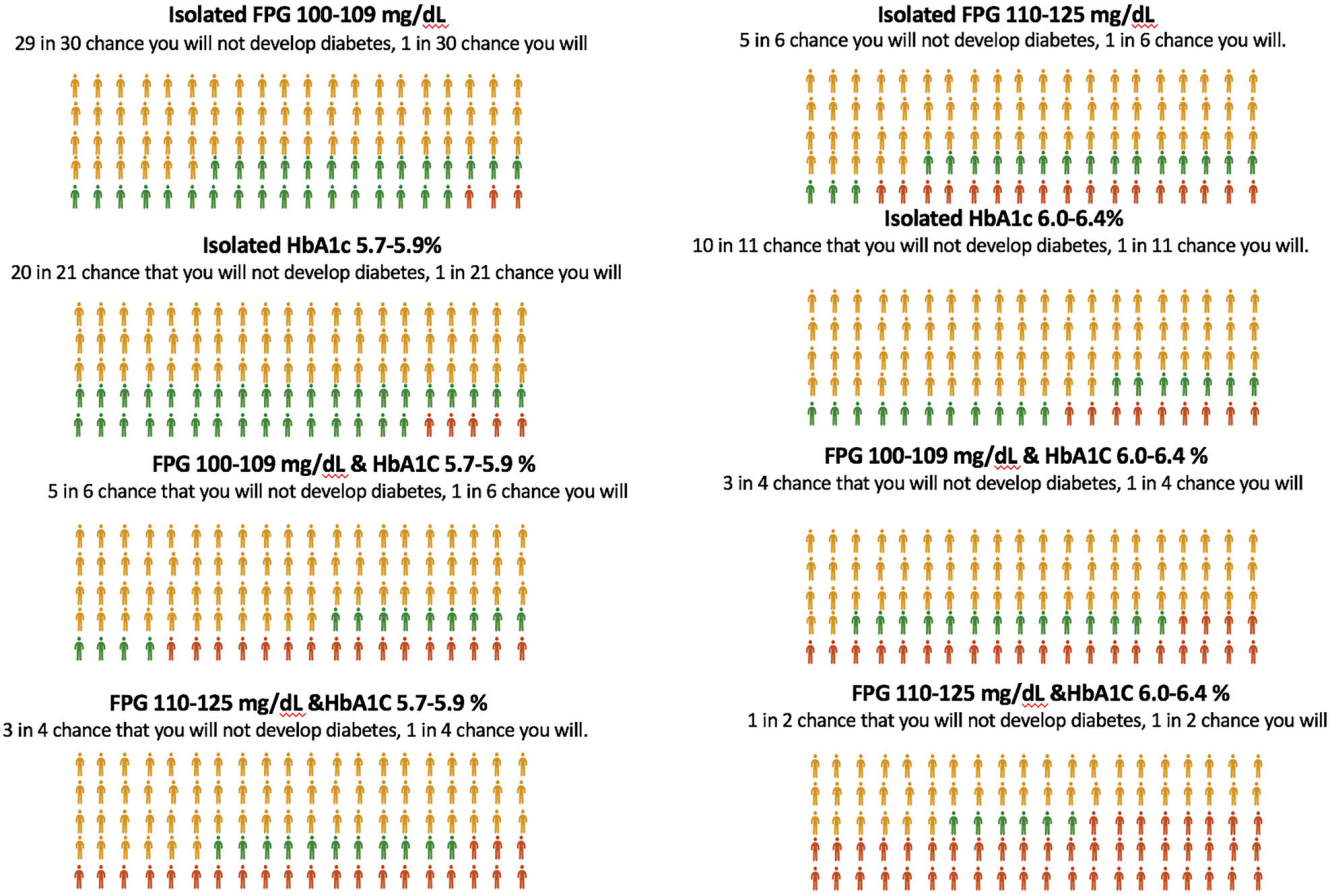
Pictograms to explain the prognosis of prediabetes per 100 people at five years’ follow-up in each prediabetes category in the PREDAPS study. Probability of diabetes non-onset versus onset, and number of people who revert to normoglycemia, continue with prediabetes , and who develop diabetes .

For each category of prediabetes, Figure 1 shows the odds against diabetes onset (converted in probability) as well as the number of participants whose blood glucose and/or HbA1c values remained at prediabetic levels, normalized, or progressed to diabetic levels during follow-up. Of every 30 individuals with isolated FPG 100–109 mg/dL and of every 2 individuals in the FPG 110–125 mg/dL plus HbA1c 6.0–6.4% group, 29 and 1 did not develop diabetes, respectively. For every 100 subjects in the first group 97 individuals did not develop diabetes and 31 normalized the glycemia, while in the second group the figures were 52 and 6, respectively.

Participants’ health-related behavior, whether at baseline or over follow-up, barely affected the progression to diabetes onset or reversion to normoglycemia according to prediabetes categories (Supplementary tables 2 and 3).

## Discussion

### Main findings

The evolution of participants with prediabetes over five-years follow-up was heterogeneous, depending on baseline FPG and HbA1c levels. The probability of diabetes non-onset/onset ranged from 97%/3%, in people categorized as prediabetic based on the sole criterion of isolated FPG levels of 100–109 mg/d-L, to 52%/48% in those whose FPG values were 110-125 mg/dL and whose HbA1c levels were 6.0–6.4%. As a result, the odds against diabetes onset were 29:1 in the former group and 1:1 in the latter, while the probability of reverting to normoglycemia was 31.2% and 6%, respectively.

### Strengths and limitations

The long period of follow-up and the annual assessments of FPG and HbA1c are strengths of this study. In contrast to other studies reporting diabetes incidence only as a rate (making it difficult for clinicians to communicate the prognosis to prediabetic patients), in the present study, the prognosis was calculated according to different prediabetes categories as a probability. Also the probability of diabetes has been compared with the probability of diabetes non-onset, which is of great interest to physicians and patients when deciding the alternatives of action. Likewise, the inclusion of probability of reversion to normoglycemia is very useful to weigh decision-making.

In most studies, there are certain overlaps in the criteria used to define prediabetes categories. In this study, the prognosis was estimated at different FPG and HbA1c thresholds, using mutually exclusive and collectively exhaustive categories. PREDAPS did not include participants with prediabetes diagnosed based on oral glucose tolerance test (OGTT) [19,22], therefore it was not possible to estimate the prognosis for different categories according to this criterion. Although OGTT values even in isolated form have been shown to predict diabetes, it is not a test performed routinely in clinical practice. Therefore, its relevance is more for scientific practice than for routine clinical practice.

The results could be biased patients who abandoned were different from those who remained. However, the distribution and frequency of age, sex, lifestyles and health problems at baseline, shown in Supplementary table 1, were similar in the subjects who completed the study than in those who abandoned it throughout the follow-up.

The present study shows the prognosis in subjects with prediabetes aged 35 to 74 years. The findings could have been different if the lower age limit had been higher. However, no modification was observed in the relationship between the different categories of prediabetes analyzed and the prognostic results, after performing separate analyzes in subjects under 50 and in subjects 50 years of age and older.

This observational study was carried out in routine clinical practice. Therefore, a complete absence of intervention cannot be ruled out, as physicians advised patients on lifestyle changes based on clinical and laboratory parameters. In any case, the impact on the development of diabetes was small since the incidence rate hardly varied throughout the five years of follow-up. Its magnitude in the first and last year was 4.1 and 4.2 per 100 person-years, respectively.

In subjects with prediabetes, other factors, apart from FPG and/or HbA1c levels, are relevant for clinical decision-making. Like social context, housing stability, and financial barriers. Such factors have not been considered in the present study. Physicians, especially in primary health care, must weigh the knowledge of these factors in each patient with the information on the different results of the evolution of prediabetes shown here.

The present study did not consider other clinical disorders and health conditions, as for example cardiovascular events, for which people with prediabetes could be at risk, and which should be communicated to patients. In the PREDAPS study, the probability of experiencing any cardiovascular event was 4.9% in the participants with prediabetes, suggesting that the incidence in the lower-risk categories was modest [23].

Finally, the analyses did not take into account the use of antihypertensive or lipid lowering drugs. The frequency of use of these drugs was high given the high prevalence of hypertension and dyslipidemia in these subjects. However, the inclusion of these conditions in the analysis did not modify the relationship between the different categories of prediabetes and the prognostic results (supplementary tables 2 and 3).

### Implications for clinical practice

The proportion of people with prediabetes who develop diabetes or return to normoglycemic levels varies in the literature [11]. The diverse criteria used to define prediabetes and the different periods of follow-up explain some of this heterogeneity, while the variable distribution of prediabetes phenotypes from one place to another also need to be considered. For example, about 20% of people with prediabetes in the Japanese population presented altered levels of both FPG and HbA1c [10,24], compared to 50% in the PREDAPS study [19,22], a difference that could explain the higher five-year incidence of diabetes in the Spanish compared to the Japanese cohort. However, the prognosis was similar in both populations when analyzed according to prediabetes phenotypes [10,19,24], suggesting that our results are probably quite similar to other geographical settings.

In observational studies following people with prediabetes for 1 to 10 years, most do not develop diabetes, and a large proportion returned to normoglycemia [9,11]. In the present study, the probability of diabetes non-onset was 80.7%, while the probability of reverting to normoglycemia was 19.7%. Other studies with similar follow-up periods have reported similar results [10,11,24,25]. These results have prompted some authors to consider that the ADA-established thresholds of 100 mg/dL for glycemia and 5.7% for HbA1c for defining prediabetes favors overdiagnosis and confers an unnecessary burden on the health system, as it converts healthy people into patients [16–18]. On the other hand, other authors argue that these values increase the diabetes onset risk, and they consider that physicians should advise weight loss and physical activity, with or without medication, to all people with abnormally high indicators in order to delay or prevent the development of diabetes [13–14]. Some authors consider it necessary to recommend behavioral modifications even in people with FPG or HbA1c values below these cutoffs [13,14,26].

The ADA thresholds for defining prediabetes were established as the result of an intersubjective consensus among numerous actors from different institutions and professional groups. Controversy around the exact values is likely to continue given the heterogeneity in the prognosis for prediabetes in different studies [27] as well as the different criteria applied in clinical practice [28]. Some physicians consider that the Hippocratic Oath requires intervention to attend people who are sick, whereas others interpret it to include care for healthy people, even if most will not develop the disease. In light of this debate, shared decision-making between people with prediabetes and their physicians could be the appropriate option for the best clinical practice, as part of win-win philosophy.

For example, doctors who recommend intervention in people with an FPG of 100–109 mg/dL and/or HbA1c of 5.7–5.9% consider that these people will benefit from preventive care. According to one study, behavioral modification in such populations can reduce the relative risk of developing diabetes to a similar extent as in people with higher values [29]. However, considering that the implementation of these measures is beneficial implies a value judgement.

According to the findings found here, 97% of people diagnosed with prediabetes based solely on FPG values, and 95% diagnosed based on HbA1c, will not develop diabetes within five years. Most of them would not consider it beneficial to attempt to increase their chances of not developing diabetes through behavioral modifications. This is despite the fact that reversion to normoglycemia could increase from 31–35% to 45%, in line with results from a meta-analysis of intervention studies that combined changes in diet and physical activity, yielding a relative risk of reversion of 1.53 [30]. Something different happens in people who belong to other categories of prediabetes. For example, 52% of the people with FPG of 110–125 mg/dL plus HbA1c of 6.0–6.4% will not develop diabetes. Considering the relative risk of diabetes incidence of 0.59 obtained in the previously mentioned meta-analysis [30], this percentage would increase to 71% with behavioral modifications. Thus, these people would likely consider this potential increase to be of great value.

Authors have also taken a public health perspective to support the appropriateness of interventions in low-risk individuals, as this contributes to reducing diabetes incidence in the population [31]. However, it is unreasonable to attribute efficiency to the option of dedicating societal resources to ensure adherence to healthy behaviors in people whose risk of developing diabetes is less than 5%. Although some studies justify the ADA cutoffs as cost-effective, this research has not specifically looked at the cost-effectiveness of interventions in the prediabetes categories associated with a lower risk of developing the disease [32–34].

Implementing preventive interventions in healthy people –with doubtful clinical justification- is quite different from treating a patient who has sought help for a specific problem [35]. This task requires physicians to improve their understanding and ability to communicate about the risk of the disease they seek to prevent and the absolute risk reduction that the possible intervention entails [36]. Physicians also need adequate ways to present the information to ensure its usefulness during clinical decision-making.

The first step should be to transmit information about the natural history of the disease. The number absolutes shown here in pictograms constitute a simple way for people with diverse levels of prediabetes indicators to understand their chances of developing diabetes, returning to normal glycemic levels, or continuing in the same prediabetic situation. Moreover, the information about the probabilities against diabetes onset can provide valuable information for subjective judgements about whether or not to adopt the preventive measures that their doctors proposals. On hearing this information, some people with prediabetes may not even want to know about the different options their physicians describes.

A second stage would provide physicians with the existing evidence supporting the intervention(s) that they recommend. In this case, they have no choice but to try to make these recommendations compatible with the odds of probabilities and the absolute numbers. However, most intervention studies about behavioral changes and/or drug prescriptions do not disaggregate the results according to various categories of prediabetes.

In summary, judgements and values associated with interpreting findings from scientific knowledge about prediabetes and its application to clinical practice vary among different physicians. Moreover, these value judgements may be different from their patients’ preferences. Communicating information on the prognosis of prediabetes in different ways that are understandable could be useful for assisting people with prediabetes and their physicians make informed and shared decisions about different intervention options.

## Supplementary Material

Supplemental Material

Supplemental Material

Supplemental Material

## PREDAPS study group

Margarita Alonso (CS De la Eria, Asturias), Beatriz Álvarez (CS Andrés Mellado, Madrid), Fernando Álvarez (CS La Calzada 2, Asturias), J Carlos Álvarez (CS Eras de Renueva, León), M^a^ del Mar Álvarez (CS Hereza, Madrid), J Joaquín Antón (CS Murcia-Centro, Murcia), Oriol Armengol (EAP Poblenou, Barcelona), Sara Artola (CS José Marvá, Madrid), Luis Ávila (Consultorio Almachar, Málaga), Carmen Babace (CS Rodríguez Paterna, La Rioja), Lourdes Barutell (CS Andrés Mellado, Madrid), M^a^ Jesús Bedoya (CS Hereza, Madrid), Belén Benito (EAP Raval Sud, Barcelona), Beatriz Bilbeny (EAP Raval Sud, Barcelona), Marti Birules (EAP Poblenou, Barcelona), Concepción Blanco (CS Sada, A Coruña), M^a^ Isabel Bobé (EAP La Mina, Barcelona), Carmen Boente (CS Porriño, Pontevedra), Antonia Borras (CS Canal Salat, Baleares), Remei Bosch (EAP Girona 2, Girona), M^a^ Jesús Brito (CS La Matanza, Baleares), Pilar Buil (EAP Azpilagaña, Navarra), J José Cabré (EAP Reus-1, Tarragona), Ainoha Cambra (CS Arrabal, Zaragoza), Francisco Carbonell (CS Mislata, Valencia), Francisco Carramiñana (CS San Roque, Badajoz), Lourdes Carrillo (CS La Victoria de Acentejo, Santa Cruz de Tenerife), Ana Casorrán (CS Fuente de San Luis, Valencia), Rafael Colas (CS Santoña, Cantabria), Blanca Cordero (CS Sta. María de Benquerencia, Toledo), Xavier Cos (EAP Sant Martí de Provençals, Barcelona), Gabriel Cuatrecasas (CAP de Sarrià, Barcelona), Cristina De Castro (CS Sta. María de Benquerencia, Toledo), Manuel De la Flor (CS Ntra. Sra. de Gracia, Sevilla), Carlos De la Sen (Consultorio San Gabriel, Alicante), Rosa Mar De Miguel (EAP Pubillas Casas, Barcelona), A María De Santiago (Unidad Docente de Atención Familiar y Comunitaria, Guadalajara), Mercedes Del Castillo (CS Andrés Mellado, Madrid), M^a^ Carmen Durán (CS Lavadores Vigo, Pontevedra), Patxi Ezkurra (CS Zumaia, Guipúzcoa), Paula Gabriel (EAP Badia del Vallès, Barcelona), Javier Gamarra (CS Medina del Campo Rural, Valladolid), Francisco García (CS Don Benito Este, Badajoz), Luis García-Giralda (CS Murcia Centro, Murcia), F Javier García-Soidán (CS Porriño, Pontevedra), M^a^ Teresa Gijón (CS Los Yébenes, Madrid), Albert Goday (Endocrinología Hospital del Mar, Barcelona), Ángel Gómez (CS Lasarte, Guipúzcoa), María del Carmen Gómez (CS Vélez-Málaga Norte, Málaga), J Carles González (Girona 3, Girona), María González (CS Alcantarilla Sangonera, Murcia), Esteban Granero (CS Vista Alegre Murcia, Murcia), Ángela Trinidad Gutiérrez (CS El Calero, Las Palmas), Félix Gutiérrez (CS Bombarda-Monsalud, Zaragoza), Luisa Gutiérrez (CS Beraun, Guipúzcoa), M Ángel Gutiérrez (CS Ávila Sur Oeste, Ávila), Ana M^a^ Hernández (CS El Calero, Las Palmas) , Mercedes Ibáñez (CS Vandel, Madrid), Rosario Iglesias (CS Lain Entralgo, Madrid), Dimas Igual (CS Manuel Encinas, Cáceres), Jaime Innenaraty (CS Hereza Leganes, Madrid), Jaime Iriarte (CS Aizarnazabal-Getaria, Guipúzcua), Ángeles Jurado (CS Salvador Caballero, Granada), Rafael Llanes (Villanueva de la Cañada, Madrid), Flora López (EAP Martorell, Barcelona), Regina López (EAP El Carmel, Barcelona), Riánsares López (CS Artilleros, Madrid), Ángela Lorenzo (CS Alcalá de Guadaira, Madrid), Carmen Losada (UGC Adoratrices, Huelva), Ramón Macía (CS Roces Montevil, Asturias), Fernando Malo (CS Ares, A Coruña), José Mancera (CS Ciudad Jardín, Málaga), M^a^ José Mansilla (CS Martín de Vargas, Madrid), M^a^ Teresa Marín (CS General Ricardos, Madrid), José Luis Martín (CS Salvador Caballero, Granada), Ana Martínez (EAP El Carmel, Barcelona), F Javier Martínez (CS Federica Monseny, Madrid), Juan Martínez (CS Yecla, Murcia), M^a^ del Carmen Martínez (EAP Raval Sud, Barcelona), Rosario Martínez (CS Oñati, Guipúzcoa), Anna Massana (EAP Raval Sud), M^a^ Soledad Mayayo (CS Martín de Vargas, Madrid), J Javier Mediavilla (CS Burgos Rural, Burgos), Luis Mendo (CS Cadreita, Navarra), J Manuel Millaruelo (CS Torrero La Paz, Zaragoza), Alicia Monzón (CS Vecindario, Las Palmas) , Ana Moreno (CAP San Roque, Badajoz), Pedro Muñoz (Unidad Docente de Medicina Familiar y Comunitaria, Cantabria), Xavier Mundet (EAP Carmel, Barcelona), Teresa Mur (CAP Terrasa Sud, Barcelona), Emma Navarro (CS Añaza, Santa Cruz de Tenerife), Jorge Navarro (CS Salvador Pau, Valencia), Pedro Nogales (CS Las Águilas, Madrid), J Carlos Obaya (CS Chopera, Madrid), Cristina Oria (CS Aizarnazabal-Guetaria, Guipúzcoa), Francisco Javier Ortega (CS Campos-Lampreana, Zamora), Francisca Paniagua (CS Ciudad Jardín, Málaga), José Luis Pardo (CS Orihuela I, Alicante), Francisco Carlos Pérez (CS Martín de Vargas, Madrid), Pedro P Pérez (CS Mallen, Sevilla), Neus Piulats (EAP Raval Sud, Barcelona), Raquel Plana (CS Ponteareas, Pontevedra), Nuria Porta (CAP Terrassa Sud, Barcelona), Santiago Poveda (CS Jumilla, Murcia), Luis Prieto (CS Cáceres-La Mejostilla, Cáceres), Ramón Pujol (EAP Tremp, Lleida), Sol Reixa (CS Arrabal, Zaragoza), Jazmín Ripoll (CS Fuente de San Luis, Valencia), Antonio Rodríguez (EAP Anglès, Girona), J José Rodríguez (CS Villaviciosa de Odón, Madrid), M^a^ Angeles Rollán (CS Los Yébenes, Madrid), M^a^ Teresa Rollán (CS Hereza, Madrid), Laura Romera (EAP Raval Nord, Barcelona), Pilar Roura (EAP Badia de Vallès, Barcelona), Jóse Félix Rubio (CS Lasarte, Guipúzcua), Irene Ruiz (EAP La Torrassa, Barcelona), Manuel Antonio Ruiz (CS Agost, Alicante), Isabel Sáenz (CS Espronceda, Madrid), Julio Sagredo (CS Los Rosales, Madrid), Alejandro Salanova (CS Fuente de San Luis, Valencia), L Gabriel Sánchez (CS Carballeda, Zamora), Manuel Sánchez (CS Vista Alegre Murcia, Murcia), Gloria Sanz (CS San José centro, Zaragoza), Mateu Seguí (UBS Es Castell, Baleares), Josefina Servent (EAP El Carmel, Barcelona), Dulce Suárez (CS El Calero, Las Palmas) , Eduard Tarragó (EAP Bellvitge, Barcelona), Jesús Torrecilla (CS Bombarda-Monsalud, Zaragoza), José Luis Torres (CS Rodríguez Paterna, La Rioja), Merè Villaró (CAP Terrassa sud, Barcelona), Carmen Yuste (EAP Badia del Vallès, Barcelona).

Guarantor’s: E. Regidor is the guarantor of this work and, as such, had full access to all the data in the study and takes responsibility for the integrity of the data and the accuracy of the data analysis.
