# Supplementary material for: Classifying and communicating risks in prediabetes according to fasting glucose and/or glycated hemoglobin: PREDAPS cohort study
Source: Scand J Prim Health Care. 2021 Aug 4;39(3):355–63. doi: 10.1080/02813432.2021.1958497 (PMC8475112; doi:10.1080/02813432.2021.1958497)
Supplement: Supplementary file 1 [file IPRI_A_1958497_SM8081.docx]

Supplementary table 3. Hazard ratio (95% confidence interval) of regression to normoglycemia according to prediabetes category

Prediabetes category	Model 1		Model 2		Model 3	
Isolated FPG 100-109 mg/dL	2.15	(1.60-2.89)	2.12	(1.57-2.87)	2.34	(1.74-3.17)
Isolated FPG 110-125 mg/dL	1.19	(0.75-1.88)	1.13	(0.71-1.81)	1.08	(0.68-1.71)
Isolated HbA1c 5.7%-5.9%	2.52	(1.94-3.27)	2.52	(1.92-3.32)	2.69	(2.06-3.50)
Isolated HbA1c 6.0%-6.4%	0.86	(0.53-1.41)	0.83	(0.51-1.36)	0.87	(0.53-1.41)
FPG 100-109 mg/dL + HbA1C 5.7%-5.9%	0.91	(0.59-1.40)	0.91	(0.59-1.42)	0.99	(0.64-1.55)
FPG 100-109 mg/dL + HbA1C 6.0%-6.4%	0.59	(0.34-1.01)	0.60	(0.35-1.04)	0.64	(0.37-1.10)
FPG 110-125 mg/dL + HbA1C 5.7%-5.9%	0.72	(0.46-1.14)	0.76	(0.48-1.20)	0.90	(0.57-1.41)
FPG 110-125 mg/dL + HbA1C 6.0%-6.4%	0.47	(0.25-0.88)	0.48	(0.26-0.90)	0.30	(0.16-0.55)
Overall	1.00		1.00		1.00	

CI: confidence interval

Model 1: Adjusted by sex and age.
Model 2: Model 1 adjusted by hypertension, low HDL levels, alcohol consumption, tobacco consumption, BMI, abdominal obesity, physical activity, compliance with breakfast, and eating fruits and vegetables daily.
Model 3: Model 1 adjusted by hypertension, low HDL levels, alcohol consumption, tobacco consumption, BMI, abdominal obesity, physical activity, compliance with breakfast, and eating fruits and vegetables daily, considered as time dependent variables.
