# Supplementary material for: Classifying and communicating risks in prediabetes according to fasting glucose and/or glycated hemoglobin: PREDAPS cohort study
Source: Scand J Prim Health Care. 2021 Aug 4;39(3):355–63. doi: 10.1080/02813432.2021.1958497 (PMC8475112; doi:10.1080/02813432.2021.1958497)
Supplement: Supplementary file 2 [file IPRI_A_1958497_SM8082.docx]

Supplementary table 2. Hazard ratio (95% confidence interval) or type 2 diabetes according to prediabetes category

Prediabetes category	Model 1		Model 2		Model 3	
Isolated FPG 100-109 mg/dL	0.22	(0.10-0.48)	0.24	(0.11-0.52)	0.24	(0.11-0.51)
Isolated FPG 110-125 mg/dL	1.32	(0.85-2.05)	1.37	(0.87-2.14)	1.37	(0.87-2.13)
Isolated HbA1c 5.7%-5.9%	0.33	(0.19-0.59)	0.38	(0.21-0.67)	0.37	(0.21-0.66)
Isolated HbA1c 6.0%-6.4%	0.72	(0.42-1.25)	0.71	(0.41-1.23)	0.72	(0.42-1.25)
FPG 100-109 mg/dL + HbA1C 5.7%-5.9%	1.00	(0.65-1.55)	0.98	(0.63-1.50)	0.97	(0.63-1.50)
FPG 100-109 mg/dL + HbA1C 6.0%-6.4%	1.66	(1.32-2.63)	1.53	(1.04-2.27)	1.52	(1.03-2.25)
FPG 110-125 mg/dL + HbA1C 5.7%-5.9%	1.86	(1.32-2.63)	1.80	(1.27-2.55)	2.05	(1.45-2.91)
FPG 110-125 mg/dL + HbA1C 6.0%-6.4%	4.52	(3.48-5.88)	4.26	(3.26-5.54)	3.79	(2.90-4.94)
Overall	1.00		1.00		1.00	

CI: confidence interval

Model 1: Adjusted by sex and age.
Model 2: Model 1 adjusted by hypertension, low HDL levels, alcohol consumption, tobacco consumption, BMI, abdominal obesity, physical activity, breakfast eating, and daily intake of fruits and vegetables.
Model 3: Model 1 adjusted by hypertension, low HDL levels, alcohol consumption, tobacco consumption, BMI, abdominal obesity, physical activity, breakfast eating, and daily intake of fruits and vegetables, considered as time-dependent variables.
