# Supplementary material for: Classifying and communicating risks in prediabetes according to fasting glucose and/or glycated hemoglobin: PREDAPS cohort study
Source: Scand J Prim Health Care. 2021 Aug 4;39(3):355–63. doi: 10.1080/02813432.2021.1958497 (PMC8475112; doi:10.1080/02813432.2021.1958497)
Supplement: Supplementary file 3 [file IPRI_A_1958497_SM8089.docx]

Supplementary table 1. Baseline participant characteristics, by prediabetes category. PREDAPS Study

Characteristics[1]	Isolated FPG 100-109 mg/dL		Isolated FPG 110-125 mg/dL		Isolated HbA1c 5.7-5.9%		Isolated HbA1c 6.0-6.4%		FPG 100-109 mg/dL & HbA1C 5.7-5.9 %		FPG 100-109 mg/dL & HbA1C 6.0-6.4 %		FPG 110-125 mg/dL &HbA1C 5.7-5.9 %		FPG 110-125 mg/dL &HbA1C 6.0-6.4 %	
	N	%	N	%	N	%	N	%	N	%	N	%	N	%	N	%
**Gender**																
Men	97	63.8	59	57.8	88	43.1	38	33.9	68	49.3	63	49.2	87	62.1	95	45.7
Women	55	36.2	43	42.2	116	56.9	74	66.1	70	50.7	65	50.8	53	37.9	113	54.3
**Age (years)**																
30-49	39	25.7	19	18.6	38	18.6	17	15.2	22	15.9	9	7.0	19	13.6	24	11.5
50-59	45	29.6	35	34.3	67	32.8	28	25.0	43	31.2	31	24.2	55	39.3	57	27.4
60-69	53	34.9	38	37.3	78	38.2	44	39.3	57	41.3	61	47.7	48	34.3	95	45.7
70-74	15	9.9	10	9.8	21	10.3	23	20.5	16	11.6	27	21.1	18	12.9	32	15.4
**Hypertension**																
No	55	36.2	34	33.3	101	49.5	36	32.1	39	28.3	33	25.8	43	30.7	50	24.0
Yes	97	63.8	68	66.7	103	50.5	76	67.9	99	71.7	95	74.2	97	69.3	158	76.0
**Low HDL levels**																
No	119	78.3	80	78.4	159	77.9	89	79.5	103	74.6	99	77.3	105	75.0	144	69.2
Yes	33	21.7	22	21.6	45	22.1	23	20.5	35	25.4	29	22.7	35	25.0	64	30.8
**Abdominal obesity**																
No (<88/102 cm)	68	44.7	38	37.3	92	45.1	30	26.8	38	27.5	26	20.3	47	33.6	52	25.0
Yes (>88/102 cm)	84	55.3	64	62.7	112	54.9	82	73.2	100	72.5	102	79.7	93	66.4	156	75.0
**General obesity**																
<30 kg/m^2	108	71.1	63	61.8	136	66.7	72	64.3	64	46.4	55	43.0	74	52.9	103	49.5
>30 kg/m^2	44	28.9	39	38.2	68	33.3	40	35.7	74	53.6	73	57.0	66	47.1	105	50.5
**Physical activity**																
Follow recommendations	62	40.8	42	41.2	85	42.1	49	43.8	68	49.3	59	46.1	58	41.4	106	51.0
Do not follow recommendations	90	59.2	60	58.8	117	57.9	63	56.2	70	50.7	69	53.9	82	58.6	102	49.0
**Smoking**																
Never smoker	62	40.8	35	34.3	82	40.2	56	50.0	75	54.3	67	52.3	57	40.7	98	47.1
Ex-smoker	65	42.8	52	51.0	76	37.3	37	33.0	44	31.9	43	33.6	63	45.0	75	36.1
Current smoker	25	16.4	15	14.7	46	22.5	19	17.0	19	13.8	18	14.1	20	14.3	35	16.8
**Alcohol consumption**																
Non-drinker	14	9.2	5	4.9	20	9.8	12	10.7	10	7.2	8	6.2	9	6.4	22	10.6
Former drinker	29	19.1	15	14.7	49	24.0	35	31.2	31	22.5	39	30.5	31	22.1	56	26.9
Drinker (occasional/daily)	109	71.7	82	80.4	135	66.2	65	58.0	97	70.3	81	63.3	100	71.4	130	62.5
**Breakfast**																
Yes	145	95.4	94	92.2	201	98.5	109	97.3	135	97.8	128	100.0	130	92.9	197	94.7
No	7	4.6	8	7.8	3	1.5	3	2.7	3	2.2	0	0.0	10	7.1	11	5.3
**Fruits**																
Yes	117	77.0	73	71.6	154	75.5	90	80.4	111	80.4	103	80.5	112	80.0	161	77.4
No	35	23.0	29	28.4	50	24.5	22	19.6	27	19.6	25	19.5	28	20.0	47	22.6
**Vegetables**																
Yes	87	57.2	60	58.8	115	56.4	62	55.4	84	60.9	79	61.7	75	53.6	111	53.4
No	65	42.8	42	41.2	89	43.6	50	44.6	54	39.1	49	38.3	65	46.4	97	46.6

FPG: fasting plasma glucose; HbA1c: glycated hemoglobin A1c.

1. Hypertension was defined as systolic blood pressure ≥140 mmHg and/or diastolic blood pressure ≥90 mmHg, being treated with antihypertensive drugs or previous diagnosis of hypertension. Low high-density lipoprotein cholesterol (HDL-c) as <40 mg/dL in men or <50 mg/dL in women. Abdominal obesity as waist circumference ≥102 cm in men and ≥88 cm in women. General obesity was defined as body mass index ≥30 kg/m2 . Physical activity was classified according to WHO recommendation: people followed the recommendations if they practiced more than 150 minutes per week of moderate aerobic physical activity, more than 75 minutes each week of vigorous aerobic physical activity or an equivalent combination. Tobacco consumption was classified as current smokers, ex-smokers and non-never smokers. Alcohol consumption was classified as drinkers, former drinkers and non-drinkers. Participants were asked to describe their usual breakfast, with a wide variety of foods being included as possible replies. Participants were dichotomized into two groups, those who did not eat breakfast or had an incomplete breakfast (e.g., only coffee, milk, chocolate, cocoa or yogurt), and the rest. Based on the data collected on the frequency of consumption of fruit and vegetables, participants were dichotomized as those who ate these foods daily, and those who ate them less often.
